# Supplementary material for: Resolving the full spectrum of human genome variation using Linked-Reads
Source: Genome Res. 2019 Apr;29(4):635–45. doi: 10.1101/gr.234443.118 (PMC6442396; doi:10.1101/gr.234443.118)
Supplement: Supplemental Material [file supp_gr.234443.118_Supplemental_longranger-2.2.zip › longranger-2.2.tar.bz2!/lr2.2-public/lib/stan/cmdstan/stan/doc/api/html/classstan_1_1mcmc_1_1stepsize__adaptation.html]

Stan: stan::mcmc::stepsize\_adaptation Class Reference


|  |  |
| --- | --- |
|  | Stan  2.5.0  probability, sampling & optimization |


- Main Page
- Related Pages
- Namespaces
- Classes
- Files

- Class List
- Class Index
- Class Hierarchy
- Class Members

All Classes Namespaces Files Functions Variables Typedefs Enumerations Enumerator Friends Macros Pages

- stan
- mcmc
- stepsize\_adaptation

Public Member Functions |
Protected Attributes |
List of all members

stan::mcmc::stepsize\_adaptation Class Reference

`#include <stepsize_adaptation.hpp>`

Inheritance diagram for stan::mcmc::stepsize\_adaptation:

|  |  |
| --- | --- |
| Public Member Functions | |
|  | stepsize\_adaptation () |
|  | |
| void | set\_mu (double m) |
|  | |
| void | set\_delta (double d) |
|  | |
| void | set\_gamma (double g) |
|  | |
| void | set\_kappa (double k) |
|  | |
| void | set\_t0 (double t) |
|  | |
| double | get\_mu () |
|  | |
| double | get\_delta () |
|  | |
| double | get\_gamma () |
|  | |
| double | get\_kappa () |
|  | |
| double | get\_t0 () |
|  | |
| void | restart () |
|  | |
| void | learn\_stepsize (double &epsilon, double adapt\_stat) |
|  | |
| void | complete\_adaptation (double &epsilon) |
|  | |

|  |  |
| --- | --- |
| Protected Attributes | |
| double | counter\_ |
|  | |
| double | s\_bar\_ |
|  | |
| double | x\_bar\_ |
|  | |
| double | mu\_ |
|  | |
| double | delta\_ |
|  | |
| double | gamma\_ |
|  | |
| double | kappa\_ |
|  | |
| double | t0\_ |
|  | |

## Detailed Description

Definition at line 11 of file stepsize\_adaptation.hpp.

## Constructor & Destructor Documentation

|  |  |  |  |  |
| --- | --- | --- | --- | --- |
| stan::mcmc::stepsize\_adaptation::stepsize\_adaptation | ( |  | ) |  |

inline

Definition at line 15 of file stepsize\_adaptation.hpp.

## Member Function Documentation

|  |  |  |  |  |  |
| --- | --- | --- | --- | --- | --- |
| void stan::mcmc::stepsize\_adaptation::complete\_adaptation | ( | double & | *epsilon* | ) |  |

inline

Definition at line 57 of file stepsize\_adaptation.hpp.

|  |  |  |  |  |
| --- | --- | --- | --- | --- |
| double stan::mcmc::stepsize\_adaptation::get\_delta | ( |  | ) |  |

inline

Definition at line 26 of file stepsize\_adaptation.hpp.

|  |  |  |  |  |
| --- | --- | --- | --- | --- |
| double stan::mcmc::stepsize\_adaptation::get\_gamma | ( |  | ) |  |

inline

Definition at line 27 of file stepsize\_adaptation.hpp.

|  |  |  |  |  |
| --- | --- | --- | --- | --- |
| double stan::mcmc::stepsize\_adaptation::get\_kappa | ( |  | ) |  |

inline

Definition at line 28 of file stepsize\_adaptation.hpp.

|  |  |  |  |  |
| --- | --- | --- | --- | --- |
| double stan::mcmc::stepsize\_adaptation::get\_mu | ( |  | ) |  |

inline

Definition at line 25 of file stepsize\_adaptation.hpp.

|  |  |  |  |  |
| --- | --- | --- | --- | --- |
| double stan::mcmc::stepsize\_adaptation::get\_t0 | ( |  | ) |  |

inline

Definition at line 29 of file stepsize\_adaptation.hpp.

|  |  |  |  |
| --- | --- | --- | --- |
| void stan::mcmc::stepsize\_adaptation::learn\_stepsize | ( | double & | *epsilon*, |
|  |  | double | *adapt\_stat* |
|  | ) |  |  |

inline

Definition at line 37 of file stepsize\_adaptation.hpp.

|  |  |  |  |  |
| --- | --- | --- | --- | --- |
| void stan::mcmc::stepsize\_adaptation::restart | ( |  | ) |  |

inlinevirtual

Reimplemented from stan::mcmc::base\_adaptation.

Definition at line 31 of file stepsize\_adaptation.hpp.

|  |  |  |  |  |  |
| --- | --- | --- | --- | --- | --- |
| void stan::mcmc::stepsize\_adaptation::set\_delta | ( | double | *d* | ) |  |

inline

Definition at line 20 of file stepsize\_adaptation.hpp.

|  |  |  |  |  |  |
| --- | --- | --- | --- | --- | --- |
| void stan::mcmc::stepsize\_adaptation::set\_gamma | ( | double | *g* | ) |  |

inline

Definition at line 21 of file stepsize\_adaptation.hpp.

|  |  |  |  |  |  |
| --- | --- | --- | --- | --- | --- |
| void stan::mcmc::stepsize\_adaptation::set\_kappa | ( | double | *k* | ) |  |

inline

Definition at line 22 of file stepsize\_adaptation.hpp.

|  |  |  |  |  |  |
| --- | --- | --- | --- | --- | --- |
| void stan::mcmc::stepsize\_adaptation::set\_mu | ( | double | *m* | ) |  |

inline

Definition at line 19 of file stepsize\_adaptation.hpp.

|  |  |  |  |  |  |
| --- | --- | --- | --- | --- | --- |
| void stan::mcmc::stepsize\_adaptation::set\_t0 | ( | double | *t* | ) |  |

inline

Definition at line 23 of file stepsize\_adaptation.hpp.

## Member Data Documentation

double stan::mcmc::stepsize\_adaptation::counter\_

protected

Definition at line 63 of file stepsize\_adaptation.hpp.

double stan::mcmc::stepsize\_adaptation::delta\_

protected

Definition at line 67 of file stepsize\_adaptation.hpp.

double stan::mcmc::stepsize\_adaptation::gamma\_

protected

Definition at line 68 of file stepsize\_adaptation.hpp.

double stan::mcmc::stepsize\_adaptation::kappa\_

protected

Definition at line 69 of file stepsize\_adaptation.hpp.

double stan::mcmc::stepsize\_adaptation::mu\_

protected

Definition at line 66 of file stepsize\_adaptation.hpp.

double stan::mcmc::stepsize\_adaptation::s\_bar\_

protected

Definition at line 64 of file stepsize\_adaptation.hpp.

double stan::mcmc::stepsize\_adaptation::t0\_

protected

Definition at line 70 of file stepsize\_adaptation.hpp.

double stan::mcmc::stepsize\_adaptation::x\_bar\_

protected

Definition at line 65 of file stepsize\_adaptation.hpp.

---

The documentation for this class was generated from the following file:

- src/stan/mcmc/stepsize\_adaptation.hpp


---

[ Stan Home Page ]

*© 2011–2014,
Stan Development Team.*
